# Supplementary material for: Host-Affected Body Coloration Dynamics in Perina nuda Larvae: A Quantitative Analysis of Color Variations and Endogenous Plant Influences
Source: Insects. 2025 Jul 17;16(7):728. doi: 10.3390/insects16070728 (PMC12294888; doi:10.3390/insects16070728)
Supplement: Supplementary file 1 [file insects-16-00728-s001.zip › insects-3686317-supplementary.pdf]

Table S1. Results of Detrended Correspondence Analysis (DCA) for body coloration indexes of *Perina nuda* larvae and host leaf endogenous compounds.

Axis	Gradient Length	Eigenvalue	Cumulative Variance Explained (%)
Axis 1	0.15	0.0018	49.22
Axis 2	0.09	0.0004	60.42
Axis 3	0.09	0.0002	66.52
Axis 4	0.08	0.0001	68.47
